# Comparative Study of AMMI- and BLUP-Based Simultaneous Selection for Grain Yield and Stability of Finger Millet [*Eleusine coracana* (L.) Gaertn.] Genotypes

**DOI:** 10.3389/fpls.2021.786839

**Published:** 2022-01-06

**Authors:** N. Anuradha, T. S. S. K. Patro, Ashok Singamsetti, Y. Sandhya Rani, U. Triveni, A. Nirmala Kumari, Nagappa Govanakoppa, T. Lakshmi Pathy, Vilas A. Tonapi

**Affiliations:** ^1^Agricultural Research Station, Acharya NG Ranga Agricultural University, Vizianagaram, India; ^2^Institute of Agricultural Sciences, Banaras Hindu University, Varanasi, India; ^3^Centre for Excellence in Millets (TNAU), Thiruvannamalai, India; ^4^Main Agricultural Research Station, University of Agricultural Sciences, Dharwad, India; ^5^ICAR-Sugarcane Breeding Institute, Coimbatore, India; ^6^ICAR-Indian Institute of Millets Research (IIMR), Hyderabad, India

**Keywords:** AMMI, BLUP, finger millet, simultaneous selection, culling, stability

## Abstract

Finger millet, an orphan crop, possesses immense potential in mitigating climate change and could offer threefold security in terms of food, fodder, and nutrition. It is mostly cultivated as a subsistence crop in the marginal areas of plains and hills. Considering the changes in climate inclusive of recurrent weather vagaries witnessed every year, it is crucial to select stable, high-yielding, area-specific, finger millet cultivars. Sixty finger millet varieties released across the country were evaluated over six consecutive rainy seasons from 2011 to 2016 at the Agricultural Research Station, Vizianagaram. The genotype × environment interaction (GEI) was found to be significant in the combined ANOVA. Furthermore, the Additive Main effects and Multiplicative Interaction (AMMI) analysis asserted that genotypes and the GEI effects accounted for approximately 89% of the total variation. Strong positive associations were observed in an estimated set of eleven stability parameters which were chosen to identify stable genotypes. Furthermore, Non-parametric and Parametric Simultaneous Selection indices (NP-SSI and P-SSI) were calculated utilizing AMMI-based stability parameter (ASTAB), modified AMMI stability value (MASV), and Modified AMMI Stability Index (MASI) to identify stable high yielders. Both methods had inherent difficulties in ranking genotypes for SSI. To overcome this, the initial culling [i.e., SSI with culling strategy (C-SSI)] of genotypes was introduced for stability. In the C-SSI method, the top ten genotypes were above-average yielders, while those with below-average yield were observed in NP-SSI and P-SSI methods. Similarly, the estimation of best linear unbiased prediction (BLUP)-based simultaneous selections, such as harmonic mean of genotypic values (HMGV), relative performance of genotypic values (RPGV), and harmonic mean of relative performance of genotypic values (HMRPGV), revealed that none of the top ten entries had below-average yield. The study has proven that C-SSI and BLUP-based methods were equally worthy in the selection of high-yielding genotypes with stable performance. However, the C-SSI approach could be the best method to ensure that genotypes with a considerable amount of stability are selected. The multi-year trial SSI revealed that entries Indaf-9, Sri Chaitanya, PR-202, and A-404; and VL324 and VL146 were ascertained to be the most stable high-yielding genotypes among medium-to-late and early maturity groups, respectively.

## Introduction

In a thrust to achieve food security, few crops were intensively cultivated while other neglected crops turned out to be “Orphan crops.” Small millets are the hitherto staple food for millions of people residing in arid and semiarid regions of Asian and African countries and are currently restricted to certain traditional growing areas. Increased health problems, due to changes in lifestyle, have driven people to rethink their food habits and deliberately shift toward nutritional crops, such as small millets. Finger millet [*Eleusine coracana* (L.) Gaertn.], one of the small millets, is highly nutritious in terms of fiber content, essential amino acids, calcium, and minerals (Sood et al., 2016). It is cheaper than milk and provides three times higher calcium content compared to milk, generally consumed as a calcium source (Puranik et al., 2017). Regular consumption of finger millet allows for healthy bone growth in children and prevents osteoporosis in adults. As it is rich in fiber with a low glycemic index, it is beneficial to include it in the diet of persons suffering from diabetics and other lifestyle diseases (Mitharwal et al., 2021). They are also rich in antioxidants with anticancer agents as well as high levels of methionine, lysine, and tryptophan, which are limited in other cereals. These attributes make finger millet a “super cereal” (Kumar et al., 2016). In subsistence farming, it is even used to cure illnesses, such as measles, pleurisy, pneumonia, and smallpox (Gupta et al., 2017). Undoubtedly, bringing back the neglected crops, such as finger millet is the prime concern of environmentalists and agricultural scientists due to its contribution toward biodiversity and livelihood to the poor in various parts of the world. India is the major producer of finger millet that is being cultivated in an area of 1.17 million hectares with a production of 2.00 million tons and an average productivity of 1,661 kg/ha (ASSOCHAM, 2021).

Finger millet is a drought-hardy crop that can grow with limited water resources, tolerate extremely high temperatures, and sustain in poor and degraded soils (Gupta et al., 2017). Nevertheless, it is a climate-resilient C_4_ crop with high water and nutrient use efficiency, unlike C_3_ crops that harness more nutrients (Sage and Zhu, 2011). This proves the crucial role of finger millet in food, nutrition, and economic security, and hence it should be called “Climate-smart Nutri-cereal” rather simply “Nutri-cereal” alone. Aforesaid potential benefits of finger millet have obliged the attention of researchers along with consumers and farmers.

In any crop, researchers and farmers aspire more stable and high-yielding varieties. Similarly, for finger millet, a breeder generally desires to develop a highly adaptable variety that adequately thrives in varied climatic conditions. However, it is arduous to achieve all aspects of quantity and quality of the produce. Researchers should rather focus on the development of stable high-yielding varieties specific to a target environment over the years instead of across environments as the preference of a variety may change in different climatic zones. In the recent past, it was moreover observed that the climate of a particular region varies considerably from one year to the next and poses quite a challenge to anticipate similar climatic conditions. Hence, the major task during the breeding of finger millet is to obtain high-yielding and stable varieties. Selection for high stability or adaptability is appropriate even for hilly and tribal belts of India, where the crop is usually grown in poor soils without any improved technology.

Grain yield is a complex trait and is genetically governed by many quantitative genes with small additive effects. Hence, the expression of this is generally affected by genotype, environment, and genotype × environment interaction (GEI). Understanding the GEI pattern among test entries in multi-environment trials (METs) is very crucial for plant breeders, as it complicates the selection of promising genotypes by declining the association between genotypic and phenotypic values (Ebdon and Gauch, 2002; Yan and Tinker, 2006). Modeling the GEI in METs assists in defining the phenotypic stability of the genotypes for a range of locations or a particular genotype for varied environmental conditions (Vaezi et al., 2017, 2018, 2019; Ghazvini et al., 2018). Several approaches exist to analyze genotype stability, including biplots obtained from additive main effects and multiplicative interaction (AMMI; Gauch, 1988) and genotype plus genotype × environment interaction (GGE; Yan et al., 2000; Yan and Kang, 2002) which gained popularity. Nevertheless, the primary constraint is that they are representative only when two principal components (PCs) are significant. With more significant PCs, stability cannot be satisfactorily explained by biplots. Amendments were made in due course, and AMMI-derived values, such as (1) AMMI Stability Index (ASI) and AMMI stability value (ASV) using two PCs and (2) Modified AMMI Stability Index (MASI) and Modified AMMI stability value (MASV), using all the significant PCs were explored to demonstrate the stability of genotypes more effectively. Several selection indices were developed for selecting a stable genotype with high yields, such as Kang’s Yield Stability Index (Kang, 1993), Bajpai’s Index (Bajpai and Prabhakaran, 2000), Simultaneous Selection Index (SSI; Rao and Prabhakaran, 2005), and Non-parametric Genotype Selection Index (Farshadfar, 2008), which guide the simultaneous selection of both stability and high yield using data from stability parameters and grain yield. Earlier best linear unbiased prediction (BLUP)-based simultaneous selections, such as harmonic mean of genotypic values (HMGV), relative performance of genotypic values (RPGV), and harmonic mean of relative performance of genotypic values (HMRPGV), were used similarly, for the simultaneous identification of high-yielding genotypes with stability and adaptability to adverse conditions (de Resende, 2004). This study intended to identify potential finger millet genotypes that might consistently perform throughout the crop years employing an appropriate SSI.

## Materials and Methods

### Plant Material and Experimental Site

**FIGURE 1:**
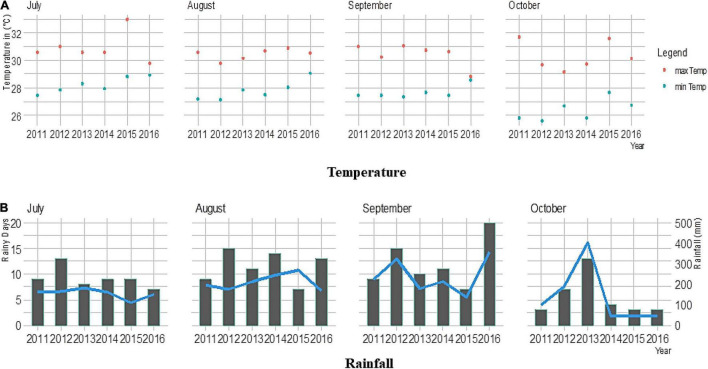
Weather parameters, including **(A)** maximum and minimum temperatures and **(B)** rainfall in the crop growing period during the main seasons of 2011–2016. The line graph represents the number of rainy days, and the bar plot shows total rainfall (in mm).

A set of 60 best performing finger millet varieties released across India were collected from different states (Table 1) of the country and evaluated during the rainy season of six consecutive crop years from 2011 to 2016 at the Agricultural Research Station, Vizianagaram Andhra Pradesh, India. The mega variety, PR 202, and another national check, GPU 67 were also included, along with the test entries. The variety VR 847 was included as a local check. Out of sixty entries, eight (i.e., Bairabhi, Chilika, Co 7, RAU 8, VL 146, VL 149, VL 324, and Champavathi) are early while the rest are medium-tolate duration. The test entries were planted in randomized block design with two replications in all the trials. Geographically, the experimental site is located at 18° 12′ N latitude and 83° 40′ E longitude at an altitude of 63 m above mean sea level comprising red sandy loam soil. Weather data, inclusive of maximum and minimum temperatures as well as rainfall, were recorded during all the six crop growing periods, as shown in Figure 1. Proper crop management practices, such as the application of a recommended dose of fertilizers (50-40-25 kg NPK/ha) and plant protection measures, were complied.

**TABLE 1:** List of finger millet genotypes evaluated under six test environments during the main seasons of 2011–2016.

Genotype code	Genotype	Genotype code	Genotype	Genotype code	Genotype
1	A 404	21	GPU 28	41	MR 1
2	Bairabhi	22	GPU 45*	42	MR 6
3	Birsa Marua 1	23	GPU 48	43	Nilochal
4	Birsa Marua 2	24	GPU 66	44	Paiyur 1
5	Chilika	25	GPU 67*	45	Paiyur 2
6	CO 10	26	Hamsa	46	PES 110
7	CO 11	27	Hima	47	Poorna
8	CO 12	28	HR 374	48	PR 202
9	TNAU 294	29	HR 911	49	PRM 1
10	TNAU 946	30	Indaf 15	50	PRM 2
11	CO 7	31	Indaf 5	51	RAU 3
12	CO 9	32	Indaf 7	52	RAU 8
13	Dapoli 1	33	Indaf 8	53	Shakti
14	Dibyasinha	34	Indaf 9	54	TRY 1
15	GN 1	35	K 7	55	VL 146
16	GN 2	36	Kalyani	56	VL 149
17	GN 3	37	KMR 204	57	VL 324
18	GN 4	38	KMR 301	58	Champavathi
19	GN 5	39	L 5	59	Bharathi
20	GPU 26	40	ML 365	60	Sri Chaitanya

*Checks included.

### Statistical Analysis

#### Additive Main Effects and Multiplicative Interaction and Best Linear Unbiased Prediction Analyses

The AMMI analysis was carried out with the grain yield data of the 60 experimental lines recorded during 6 consecutive crop years. AMMI decomposes residual matrices through singular value decomposition (SVD) after fitting main effects and also captures non-linear GEI, unlike regression models. The AMMI analysis was performed by subjecting grain yield to the following model (Gauch et al., 1996):


$${\text{Y}}_{\text{ij}}=\mu +{\text{g}}_{\text{i}}+{\text{e}}_{\text{j}}+\sum_{k=1}^{n}\lambda_{k}\alpha_{ik}\gamma_{jk}+\theta_{ij}$$

where Y_ij_ is the mean yield of the genotype *i* (*i* = 1, 2, …, 60) in the environment *j* (*j* = 1, 2, …, 6); μ is the general mean, g_i_ is the *i*th genotypic effect; e_j_ is the *j*th environment effect; λ_k_ is the eigenvalue of the principal component analysis (PCA) axis *k*; α_ik_ and γ_jk_ are the *i*th genotype in *j*th environment PCA scores for the PCA axis *k*; θ_ij_ is the residual.

#### Estimation of Stability Indices and Their Association

The AMMI-based stability parameters (ASTABs), such as averages of the squared eigenvector values (EV; Zobel, 1994), Annicchiarico’s D parameter (D; Annicchiarico, 1997), sums of absolute value of the interaction principal component (IPC) scores (SIPC; Sneller et al., 1997), ASV (Purchase, 1997), Zhang’s D parameter or AMMI statistical coefficient or AMMI distance or ASI (DZ; Zhang et al., 1998), ASTAB (Rao and Prabhakaran, 2005), sum across environments of the absolute value of GEI modeled by AMMI (AVAMGE; Zali et al., 2012), stability measure based on fitted AMMI model (FA; Zali et al., 2012), absolute value of the relative contribution of IPCs to the interaction (Za; Zali et al., 2012), ASI (Jambhulkar et al., 2014), MASI (Ajay et al., 2018b), and MASV (Ajay et al., 2019), were calculated. The Spearman’s rank correlations among all the 13 stability values were computed.

#### Simultaneous Selection Index

**FIGURE 2:**
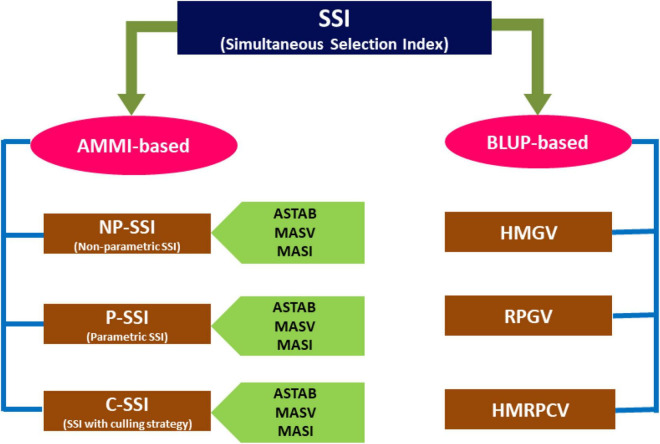
Various approaches used for the simultaneous selection of finger millet genotypes evaluated during the six main seasons of 2011–2016.

Although AMMI and GGE biplots can be considered the best tools for simultaneously visualizing the mean grain yield and genotype stability, these cannot provide the exact numerical information required for comparison. Therefore, biplots alone cannot be relied on where more than two PCs are required to interpret a considerable proportion of GEI. The stability parameters in this study, namely, ASTAB, MASI, and MASV, utilize all significant PCs for their estimation and were also considered for SSI calculation. This study comprised four approaches to estimate the SSI, where the first three methods were based on AMMI scores while the fourth relied on BLUP scores of stability (Figure 2).

##### Non-parametric Simultaneous Selection Index

This is based on the cumulative ranking of genotypes in which the top ranks were assigned to the highest grain yielding genotypes and least ranks to lowest grain yielding genotypes. In contrast, it was reversed in the case with stability, where the lowest values were assigned the top rank (Ajay et al., 2020). The two ranks were simply added and aligned in ascending order, and re-ranking was given from 1 to 60.

##### Parametric Simultaneous Selection Index

This approach is based on average yield and stability as suggested by Rao and Prabhakaran (2005), and the index was calculated using the following formula:


$$\text{P}-{\text{SSI}}_{\text{i}}=\alpha \frac{{\bar{Y}}_{i.}}{\bar{Y}..}+\beta \frac{\frac{1}{SP_{i}}}{\frac{1}{g}\sum_{1}^{g}\frac{1}{SP_{i}}}$$

where P-SSI is the SSI of the *i*th genotype; ${\bar{Y}}_{i.}$ is the mean grain yield of the *i*th genotype during six years of testing; $\bar{Y_{..}}$ is the overall mean grain yield; *SP*_*i*_ is the stability parameter value (ASTAB/MASI/MASV) of *i*th genotype; *g* is the number of genotypes evaluated. α and β are the weights attached to grain yield and stability, respectively, to arrive at an index of a genotype with a limit that sum of α and β counts to 100%. In this study, the weights of α and β were assigned 70 and 30%, respectively, giving more weight to grain yield for calculating P-SSI. Initially, the genotype with the highest P-SSI score was ranked first, followed by genotypes with descending scores, and the least score genotype was ranked 60.

##### Simultaneous Selection Indexes With Culling Strategy

In this study, we introduced a slight modification in SSI where genotypes were initially screened for stability. Only those genotypes with more than above-average stability (scores less than the mean value of stability scores) were considered to be qualified for stability or simply as stable genotypes. These qualified genotypes were arranged in descending order of their grain yield. The highest yielder was attributed the first rank while the lowest yielder attained the last rank.

##### Best Linear Unbiased Prediction-Based Stability and Adaptability

This approach involved the estimation of HMGV (to infer both yield and stability), RPGV (to investigate the mean yield and genotypic adaptability), and HMRPGV (to evaluate stability, adaptability, and yield simultaneously) through the formulae given in the study by de Resende (2004, 2016).


$${\text{HMGV}}_{\text{i}}=\frac{n}{\sum_{j=1}^{n}\left(\frac{1}{\text{G}V_{ij}}\right)}$$

$${\text{RPGV}}_{\text{i}}=\frac{1}{n}\left[\frac{\left(\sum_{j=1}^{n}{\text{GV}}_{ij}\right)}{{\text{M}}_{j}}\right]$$

$${\text{HMRPGV}}_{\text{i}}=\frac{n}{\sum_{j=1}^{n}\left(\frac{1}{{\text{RPGV}}_{ij}}\right)}$$

where *n* is the number of crop years (*n* = 6); GV_ij_ is the genetic value of *i*th genotype in *j*th year where GV_ij_ = u_j_ + g_i_ + ge_ij_, u_j_ is the average of *j*th crop year, g_i_ is the BLUP value of *i*th genotype, and ge_ij_ is the BLUP value of the interaction between *i*th genotype and *j*th crop year; M_j_ is the mean grain yield in the *j*th year.

### Software Used

All the ASTABs and correlations among the stability parameters were computed using the functions of “agricolae” (De Mendiburu, 2015) and “ammistability” (Ajay et al., 2018a) packages in R (R Core Team, 2018). The estimation of BLUP-based stability models, such as HMGV, RPGV, and HMRPGV, was performed in R using the “lme4” package (Bates et al., 2015).

## Results

### Additive Main Effects and Multiplicative Interaction Analysis

The basic statistical analysis for the grain yield data of 60 genotypes during 6 years showed that considerable variation existed among different genotypes within environment (Supplementary Table 1). The grain yield data of 60 test genotypes from 6 consecutive years were subject to combined ANOVA and AMMI analysis after confirming the homogeneity of error variance through Bartlett’s test (*p* > 0.05). Mean squares from the combined ANOVA revealed that the environments, genotypes, and GEI showed significant variation at 0.1% (*p* < 0.001) for grain yield (Table 2). The AMMI analysis recorded significant variation (*p* < 0.001) among the studied genotypes, environments, and also GEI (Table 2). Genotypes contributed a large portion of total variation (52%), whereas the environment and GEI contributed about 7.1 and 37.8%, respectively. Furthermore, the analysis revealed that GEI was significantly explained by the first four PCs. Among them, the first PC contributed 46.8% toward the total GEI while second, third, and fourth PCs contributed 28.3, 18.6, and 3.8%, respectively.

**TABLE 2:** Additive Main effects and Multiplicative Interaction (AMMI) analysis for grain yield data of 60 finger millet genotypes under six test environments during the main seasons of 2011–2016.

Source of variation	*df*	MSS	% contribution toward total variation
Environments	5	365.8***	7.1
Replication (within environment)	6	5.9***	0.1
Genotype	59	225.5***	51.8
GEI	295	32.9***	37.8

PC1	63	72.1***	46.8 of GEI
PC2	61	45.0***	28.3 of GEI
PC3	59	30.7***	18.6 of GEI
PC4	57	6.4***	3.8 of GEI
PC5	55	4.4*^ns^*	2.5 of GEI
Residuals	354	3.7	3.2

***Significant at 0.1% (p < 0.001); ns, non-significant at 5%. df, degrees of freedom; MSS, mean sum of squares; GEI, genotype × environment interaction.

*PC1, PC2, PC3, PC4, and PC5 represented the first five principal components.*

#### Additive Main Effects and Multiplicative Interaction 1 and Additive Main Effects and Multiplicative Interaction 2 Biplots

**FIGURE 3:**
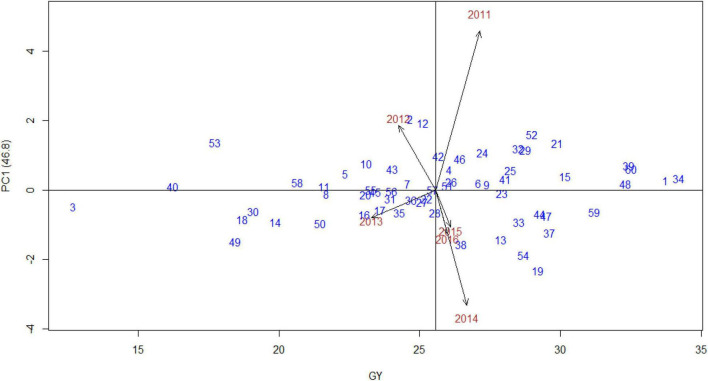
AMMI1 biplot [grain yield vs. principal component 1 (PC1)] for grain yield (00’ kg/ha) of 60 finger millet genotypes evaluated under six test environments during the main seasons of 2011–2016.

**FIGURE 4:**
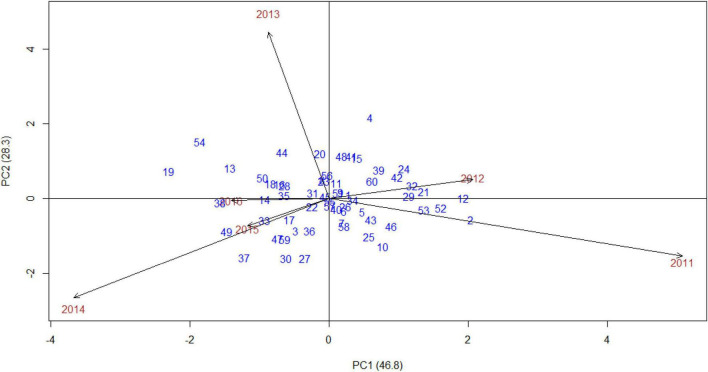
AMMI2 biplot (PC1 vs. PC2) for grain yield (kg/ha) of 60 finger millet genotypes evaluated under six test environments during the main seasons of 2011–2016.

The AMMI stability showing the relationship between experimental genotypes and test environments across different seasons was presented in “grain yield vs. PC1 scores,” i.e., AMMI1 (Figure 3). The years 2011 and 2014 were farthest from biplot origin, with long vectors representing strong interaction forces, while 2015 and 2016 were nearer to the origin and had shorter vectors with weak interaction forces. The biplot depicted that entries, such as 34 (Indaf 9), 1 (A 404), 48 (PR 202), 39 (L 5), 60 (Sri Chaitanya), and 59 (Bharathi), had maximum grain yield. In contrast, entries, namely, 3 (Birsa Marua 1), 40 (ML 365), 53 (Shakti), 49 (PRM 1), and 18 (GN 4), had poor yields, indicating their high adaptability across the seasons. The mean grain yield of all the genotypes was 2,557 kg/ha with a range of 1,268 kg/ha (Birsa Marua 1) to 3,419 kg/ha (Indaf 9). The AMMI2 biplot, which is constructed between the first two IPCs, explained 75% of the GEI (Figure 4). Entries, such as 57 (VL 324), 22 (GPU 45), 51 (RAU 3), and 26 (Hamsa), prevail near the origin in AMMI2.

### Estimation of Additive Main Effects and Multiplicative Interaction-Based Stability Indices

The visualization of AMMI biplots for grain yield was difficult since 60 genotypes were studied with many of them overlapping, creating a fuzzy figure. Various stability parameters related to AMMI analysis, such as ASI, ASV, ASTAB, AVAMGE, DA, DZ, EV, FA, MASI, MASV, SIPC, and Za, were computed and are presented in Table 3. The scores of EV were close to 0, followed by Za. Among the estimated stability statistics, ASTAB, AVAMG, DA, DZ, EV, FA, MASV, and SIPC showed similar results, and the genotypes, namely, 57 (VL 324), 22 (GPU 45), and 51 (RAU 3), were found to be highly stable according to these indices. Based on ASI and ASV, genotypes 55 (VL 146) followed by 22 (Paiyur 2) were highly stable, while ZA revealed that genotypes 57 (VL 324) followed by 55 (VL 146) had maximum stability.

**TABLE 3:** Various stability parameter estimates of 60 finger millet genotypes evaluated under six test environments during the main seasons of 2011–2016.

Genotype code	Grain yield (kg/ha)	ASI	ASV	ASTAB	AVAMGE	DA	DZ	EV	FA	MASI	MASV	SIPC	Za
1	3374	0.13	0.47	1.19	10.90	5.59	0.23	0.01	31.25	0.21	4.32	1.84	0.06
2	2466	0.97	3.42	4.81	28.49	14.81	0.33	0.03	219.22	0.97	4.36	3.37	0.19
3	1268	0.33	1.18	1.32	10.90	6.64	0.22	0.01	44.06	0.34	1.86	2.08	0.08
4	2606	0.67	2.38	6.60	33.31	15.40	0.43	0.05	237.21	0.71	7.00	4.09	0.18
5	2236	0.25	0.88	1.83	13.21	7.40	0.27	0.02	54.78	0.32	5.41	2.48	0.09
6	2708	0.14	0.48	1.84	12.97	6.05	0.33	0.03	36.59	0.19	3.58	2.33	0.06
7	2457	0.20	0.72	0.93	9.14	5.37	0.19	0.01	28.79	0.23	2.95	1.80	0.07
8	2168	0.15	0.53	0.40	7.76	3.75	0.11	0.00	14.06	0.17	1.96	1.01	0.04
9	2740	0.09	0.33	0.40	5.77	3.03	0.15	0.01	9.18	0.12	1.85	1.16	0.04
10	2309	0.51	1.81	2.69	18.48	10.14	0.27	0.02	102.73	0.53	3.98	2.85	0.14
11	2160	0.13	0.45	0.72	8.94	4.77	0.15	0.01	22.77	0.19	3.60	1.39	0.05
12	2511	0.90	3.19	3.85	26.91	13.41	0.29	0.02	179.91	0.90	3.42	2.46	0.14
13	2788	0.71	2.51	3.56	25.98	12.14	0.30	0.02	147.48	0.73	5.19	3.32	0.17
14	1987	0.43	1.54	2.67	19.55	9.27	0.32	0.03	85.86	0.48	5.74	2.82	0.11
15	3016	0.36	1.26	2.39	19.36	8.62	0.30	0.02	74.23	0.38	4.16	2.94	0.11
16	2303	0.35	1.24	1.33	14.97	7.04	0.20	0.01	49.61	0.38	4.19	2.07	0.10
17	2358	0.31	1.10	1.19	14.27	6.01	0.23	0.01	36.17	0.32	2.05	2.10	0.08
18	1870	0.41	1.45	0.97	13.64	6.48	0.15	0.01	42.04	0.41	1.94	1.68	0.09
19	2918	1.10	3.89	9.49	43.80	19.30	0.52	0.07	372.65	1.15	9.46	5.56	0.26
20	2308	0.35	1.23	2.10	15.89	7.97	0.29	0.02	63.60	0.35	2.04	2.20	0.08
21	2988	0.64	2.25	2.89	20.16	10.49	0.31	0.02	110.11	0.65	4.08	2.97	0.13
22	2524	0.13	0.46	0.14	4.12	2.23	0.07	0.00	4.97	0.13	0.58	0.68	0.03
23	2791	0.14	0.48	0.26	5.90	2.99	0.09	0.00	8.92	0.14	0.90	0.81	0.03
24	2722	0.55	1.96	3.20	18.27	10.07	0.37	0.03	101.46	0.56	3.23	3.41	0.14
25	2822	0.39	1.39	1.98	15.01	8.27	0.26	0.02	68.41	0.41	3.51	2.69	0.11
26	2613	0.13	0.45	0.42	7.22	3.42	0.13	0.00	11.68	0.15	2.21	1.24	0.04
27	2508	0.48	1.69	3.80	20.87	11.09	0.37	0.03	123.03	0.50	4.29	3.45	0.13
28	2554	0.32	1.12	2.84	17.25	8.82	0.36	0.03	77.79	0.38	5.96	3.13	0.11
29	2875	0.54	1.90	1.92	13.78	8.65	0.25	0.02	74.83	0.55	3.09	2.31	0.10
30	1909	0.54	1.90	3.78	23.74	11.70	0.33	0.03	136.82	0.56	5.02	3.38	0.15
31	2396	0.12	0.42	1.63	11.08	7.09	0.23	0.01	50.29	0.26	6.18	1.66	0.07
32	2850	0.56	2.00	4.20	24.51	12.30	0.35	0.03	151.19	0.64	8.26	3.37	0.15
33	2851	0.47	1.65	2.00	18.03	8.79	0.23	0.01	77.21	0.49	4.67	2.57	0.12
34	3419	0.16	0.55	0.56	7.59	4.29	0.13	0.00	18.42	0.20	3.27	1.17	0.05
35	2429	0.30	1.08	0.50	8.43	4.73	0.11	0.00	22.40	0.31	1.67	1.05	0.06
36	2469	0.28	0.98	0.94	12.47	5.87	0.16	0.01	34.49	0.28	2.05	1.61	0.07
37	2960	0.73	2.57	4.04	28.21	12.83	0.32	0.03	164.61	0.73	3.19	3.12	0.16
38	2646	0.73	2.59	3.72	21.51	12.39	0.31	0.02	153.45	0.76	5.92	3.06	0.15
39	3242	0.40	1.40	2.94	17.86	10.03	0.30	0.02	100.51	0.47	6.84	3.04	0.13
40	1623	0.09	0.33	0.22	4.38	2.34	0.11	0.00	5.48	0.10	0.87	0.85	0.03
41	2803	0.35	1.25	3.44	21.19	10.67	0.33	0.03	113.92	0.44	7.30	3.01	0.12
42	2566	0.48	1.71	1.30	16.58	7.61	0.17	0.01	57.85	0.48	1.96	1.75	0.10
43	2403	0.32	1.14	1.16	11.39	6.58	0.18	0.01	43.34	0.35	3.66	1.86	0.09
44	2925	0.47	1.67	2.20	15.88	9.06	0.25	0.02	82.02	0.47	2.52	2.56	0.12
45	2341	0.03	0.11	0.62	7.16	4.31	0.14	0.01	18.58	0.15	3.85	0.95	0.03
46	2643	0.47	1.66	1.62	16.29	8.13	0.20	0.01	66.11	0.48	3.03	2.29	0.11
47	2948	0.46	1.63	1.97	15.66	8.65	0.24	0.01	74.88	0.47	2.86	2.54	0.12
48	3230	0.33	1.17	1.56	11.52	7.32	0.22	0.01	53.54	0.34	2.19	1.98	0.08
49	1845	0.74	2.60	3.22	23.27	11.71	0.29	0.02	137.10	0.74	2.97	3.02	0.15
50	2145	0.48	1.68	1.57	14.17	8.00	0.21	0.01	64.01	0.48	3.11	2.31	0.11
51	2599	0.07	0.26	0.22	4.84	2.61	0.09	0.00	6.80	0.10	2.04	0.82	0.03
52	2899	0.75	2.67	3.04	23.66	11.45	0.29	0.02	131.15	0.76	2.94	2.67	0.13
53	1772	0.64	2.28	2.09	20.06	9.71	0.23	0.01	94.22	0.64	2.41	2.14	0.11
54	2868	0.97	3.43	6.10	27.23	16.13	0.38	0.04	260.15	0.98	4.78	4.09	0.22
55	2326	0.02	0.08	0.37	6.30	2.81	0.14	0.01	7.87	0.08	2.06	0.94	0.02
56	2401	0.18	0.62	0.50	7.50	4.22	0.12	0.00	17.81	0.19	1.91	1.04	0.04
57	2544	0.06	0.20	0.06	2.53	1.32	0.05	0.00	1.73	0.06	0.36	0.36	0.01
58	2065	0.23	0.82	0.72	10.11	5.04	0.15	0.01	25.44	0.24	1.87	1.47	0.06
59	3119	0.43	1.51	2.48	16.68	9.12	0.29	0.02	83.17	0.45	4.07	3.06	0.12
60	3251	0.32	1.12	1.81	15.44	6.60	0.32	0.03	43.51	0.32	1.99	2.39	0.08
Mean	**2557**	**0.40**	**1.41**	**2.14**	**15.70**	**8.04**	**0.24**	**0.02**	**78.86**	**0.43**	**3.55**	**2.27**	**0.10**

*AMGE, sum across environments of GEI modeled by AMMI; ASI, AMMI Stability Index; ASV, AMMI stability value; ASTAB, AMMI-based stability parameter; AVAMGE, sum across environments of absolute value of GEI modeled by AMMI; DA, Annicchiarico’s D parameter; DZ, Zhang’s D parameter; EV, averages of the squared eigenvector values; FA, stability measure based on fitted AMMI model; MASI, Modified AMMI Stability Index; MASV, modified AMMI stability value; SIPC, sums of the absolute value of the IPC scores; Za, absolute value of the relative contribution of IPCs to the interaction. Bold values indicated mean values.*

**FIGURE 5:**
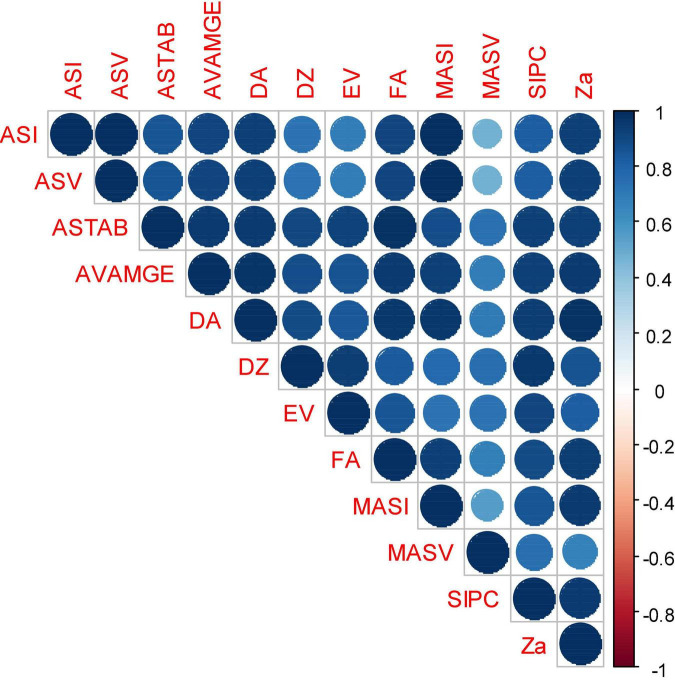
Spearman’s rank correlation among various stability and simultaneous selection indices estimated for grain yield data of 60 finger millet genotypes evaluated under six test environments. AMGE, sum across environments of genotype × environment interaction (GEI) modeled by AMMI; ASI, AMMI Stability Index; ASV, AMMI stability value; ASTAB, AMMI-based stability parameter; AVAMGE, sum across environments of the absolute value of GEI modeled by AMMI; DA, Annicchiarico’s D parameter; DZ, Zhang’s D parameter; EV, averages of the squared eigenvector values; FA, stability measure based on fitted AMMI model; MASI, Modified AMMI Stability Index; MASV, modified AMMI stability value; SIPC, sums of the absolute value of the IPC scores; Za, absolute value of the relative contribution of IPCs to the interaction.

In an attempt to reveal the relationship between each pair of AMMI stability parameters, Spearman’s rank correlations (Figure 5) revealed a strong association among the estimated AMMI-based indices. Results demonstrated a significant association for MASV with almost all the parameters, though at a relatively lesser magnitude. According to the results, ASI showed a strong correlation with most of the parameters while MASV was minimally associated with other parameters, such as ASI, ASV, and MASI (0.47, 0.47, and 0.56, respectively). It was ascertained that ASI and ASV were 100% associated. Three stability parameters, namely, ASTAB representing most of these parameters, MASV, and MASI, were considered for further analysis of SSI (Table 4). According to NP-SSI, genotypes 34 (Indaf 9) followed by 1 (A 404) were found to be highly stable based on ASTAB and MASI, while based on MASV, genotypes 60 (Sri Chaitanya), 23 (GPU 48), and 48 (PR 202) had surpassed other genotypes. Similarly, under P-SSI, ASTAB and MASI showed genotypes 57 (VL 324) and 22 (GPU 45) as the best stable with high grain yield while 57 (VL 324) followed by 55 (VL 146) had a good score of SSI based on MASI (Table 4).

**TABLE 4:** Ranking of finger millet genotypes through non-parametric, parametric, and culling methods.

Genotype code	Non-parametric cumulative rank			Simultaneous selection index-based rank			Rank after culling^†^		
			
	ASTAB	MASV	MASI	ASTAB	MASV	MASI	ASTAB	MASV	MASI
1	2	17	2	15	17	10	2	-	2
2	56	50	57	56	45	56	-	-	-
3	50	38	51	54	44	58	37	34	32
4	52	52	49	55	55	49	-	-	-
5	49	58	42	41	58	35	29	-	26
6	27	26	11	35	34	12	11	-	9
7	26	33	22	17	35	18	19	21	16
8	29	30	28	9	23	13	30	27	27
9	4	4	4	7	7	4	10	11	8
10	53	52	53	50	51	51	-	-	-
11	35	55	32	14	52	16	31	-	28
12	53	38	54	53	38	53	-	19	-
13	40	40	44	44	39	46	-	-	-
14	58	60	55	58	60	55	-	-	-
15	12	19	9	33	27	20	-	-	5
16	41	56	47	29	53	43	28	-	25
17	33	26	34	23	18	30	24	24	21
18	45	37	52	24	32	54	34	30	30
19	41	41	43	51	48	48	-	-	-
20	51	30	44	45	21	37	27	26	24
21	20	19	24	37	28	36	-	-	-
22	9	9	12	2	2	7	17	18	14
23	3	2	4	5	3	6	9	10	7
24	38	25	39	43	31	41	-	12	-
25	20	22	18	32	29	25	8	9	6
26	10	17	9	8	14	9	13	14	10
27	53	46	48	52	41	45	-	-	-
28	45	52	30	47	54	32	-	-	12
29	12	13	26	26	25	34	6	8	-
30	60	59	58	60	59	57	-	-	-
31	38	57	28	36	57	21	23	-	20
32	43	42	36	46	49	40	-	-	-
33	18	33	24	31	36	31	7	-	-
34	1	5	1	10	9	8	1	1	1
35	15	15	26	11	8	27	20	22	17
36	24	23	23	18	15	23	18	20	15
37	34	11	30	40	22	39	-	4	-
38	47	45	50	48	50	50	-	-	-
39	15	28	13	30	33	19	-	-	-
40	32	30	32	4	4	5	36	33	31
41	36	43	19	42	47	29	-	-	-
42	20	12	40	22	10	42	15	16	-
43	30	43	37	21	40	33	21	-	18
44	15	5	17	34	12	28	-	6	-
45	28	49	19	13	46	11	25	-	22
46	24	23	34	27	30	38	12	13	-
47	11	5	15	25	16	26	5	5	-
48	5	2	6	19	6	17	4	3	4
49	59	50	60	59	56	60	-	31	-
50	48	48	55	39	42	52	32	28	-
51	7	14	7	3	11	3	14	15	11
52	30	10	38	38	19	44	-	7	-
53	56	46	59	57	43	59	35	32	-
54	43	33	44	49	37	47	-	-	-
55	18	33	16	6	20	2	26	25	23
56	20	21	21	12	13	14	22	23	19
57	7	5	7	1	1	1	16	17	13
58	36	29	40	16	24	24	33	29	29
59	12	16	13	28	26	22	-	-	-
60	6	1	3	20	5	15	3	2	3

*ASTAB, AMMI-based stability parameter; MASV, modified AMMI stability value; MASI, Modified AMMI Stability Index.*

*^†^In C-SSI, genotypes were culled for stability using ASTAB, MASV, and MASI values where genotypes with scores lesser than the corresponding mean values were selected.*

*The mean values for ASTAB, MASI, and MASV are 2.14, 0.42, and 3.55, respectively.*

In the C-SSI method, ranks were allotted based on grain yield after culling genotypes with less than mean stability estimates (Table 4). The results showed that 37 out of 60 genotypes were qualified to be stable since they recorded less than the mean stability value for ASTAB (2.14) and, similarly, 34 genotypes for MASI (<0.43) and 32 genotypes for MASV (<3.55) with scores less than their representative mean values of stability.

### Estimation of Best Linear Unbiased Prediction-Based Stability Indices

The BLUP-based SSIs, such as HMGV, RPGV, and HMRPGV (Table 5), were estimated using BLUP-derived values for grain yield to check which method can be a better choice for selecting stable and high-yielding genotypes. The genotypes, namely, 34 (Indaf 9), 1 (A 404), 60 (Sri Chaitanya), 48 (PR 202), and 39 (L5), were identified as highly stable and high-yielding genotypes according to the stability parameters, such as HMGV, RPGV, and HMRPGV.

**TABLE 5:** BLUP-based ranking of 60 finger millet genotypes evaluated under six environments during the main seasons of 2011–2016.

Genotype code	Grain yield		HMGV		RPGV		HMRPGV	
	kg/ha	Rank	Score	Rank	Score	Rank	Score	Rank
1	3374	2	33.43	2	1.32	2	1.31	2
2	2466	37	23.57	42	0.96	36	0.93	40
3	1268	60	11.84	60	0.5	60	0.47	60
4	2606	28	25.32	30	1.03	26	0.98	33
5	2236	49	21.87	49	0.87	49	0.86	49
6	2708	24	26.73	23	1.06	24	1.05	23
7	2457	38	24.17	37	0.96	38	0.95	37
8	2168	50	21.72	50	0.85	51	0.85	50
9	2740	22	27.3	20	1.07	23	1.07	20
10	2309	46	21.89	48	0.9	47	0.87	48
11	2160	51	21.58	51	0.85	50	0.84	51
12	2511	34	24.2	34	0.98	34	0.95	34
13	2788	21	27.13	21	1.09	20	1.06	21
14	1987	54	18.95	54	0.78	54	0.75	54
15	3016	7	29.95	7	1.18	7	1.17	7
16	2303	48	22.66	47	0.9	48	0.89	46
17	2358	43	23.16	44	0.92	43	0.91	43
18	1870	56	18.53	55	0.74	55	0.73	55
19	2918	12	26.9	22	1.14	12	1.06	22
20	2308	47	22.94	46	0.91	45	0.89	47
21	2988	8	29.34	8	1.17	8	1.15	8
22	2524	33	25.1	32	0.99	32	0.99	29
23	2791	20	27.86	15	1.09	21	1.09	15
24	2722	23	26.72	24	1.07	22	1.04	24
25	2822	18	27.51	17	1.1	18	1.08	17
26	2613	27	25.97	25	1.02	28	1.02	25
27	2508	35	23.65	40	0.97	35	0.94	38
28	2554	31	25	33	1	31	0.98	32
29	2875	14	28.24	13	1.12	15	1.11	12
30	1909	55	17.34	57	0.74	56	0.69	56
31	2396	42	23.77	39	0.94	40	0.93	41
32	2850	17	27.71	16	1.11	16	1.09	16
33	2851	16	28.01	14	1.11	17	1.1	14
34	3419	1	33.87	1	1.33	1	1.33	1
35	2429	39	24.17	36	0.95	39	0.95	35
36	2469	36	24.2	35	0.96	37	0.95	36
37	2960	9	28.45	11	1.15	9	1.12	11
38	2646	25	25.66	28	1.04	25	1	28
39	3242	4	32.05	5	1.27	3	1.25	4
40	1623	59	16.09	59	0.64	59	0.63	59
41	2803	19	27.47	19	1.1	19	1.08	18
42	2566	30	25.41	29	1.01	30	0.99	30
43	2403	40	23.58	41	0.94	41	0.93	42
44	2925	11	29	9	1.15	10	1.13	9
45	2341	44	23.32	43	0.92	44	0.91	44
46	2643	26	25.82	27	1.03	27	1.02	26
47	2948	10	28.8	10	1.15	11	1.13	10
48	3230	5	32.07	4	1.26	5	1.25	5
49	1845	57	17.41	56	0.72	57	0.69	57
50	2145	52	21.24	52	0.84	52	0.83	52
51	2599	29	25.93	26	1.02	29	1.02	27
52	2899	13	28.32	12	1.13	13	1.11	13
53	1772	58	17.02	58	0.7	58	0.67	58
54	2868	15	27.48	18	1.13	14	1.07	19
55	2326	45	23.11	45	0.91	46	0.91	45
56	2401	41	23.96	38	0.94	42	0.94	39
57	2544	32	25.32	31	0.99	33	0.99	31
58	2065	53	20.23	53	0.81	53	0.8	53
59	3119	6	30.55	6	1.21	6	1.2	6
60	3251	3	32.25	3	1.27	4	1.26	3

*HMGV, harmonic mean of genotypic values; RPGV, relative performance of genotypic values; HMRPGV, harmonic mean of relative performance of genotypic values.*

### Top Ten Stable High-Yielding Entries

The results showed that among the top ten genotypes identified through various SSIs, few shared a commonality (Table 6 and Supplementary Table 2). In NP-SSI ASTAB, four stable genotypes, namely, Indaf 9, A 404, PR 202, and Sri Chaitanya, possessed more than 3,000 kg/ha grain yield. In contrast, in NP-SSI MASI, along with the four genotypes, GN1 was also considered in the top 10 stable high-yielding genotypes, while in NP-SSI MASV, only three genotypes (i.e., Indaf 9, PR 202, and Sri Chaitanya) recorded more than 3,000 kg/ha grain yield. The top ten genotypes in P-SSI included a very small number of genotypes with more than 3,000 kg/ha grain yield, and it was observed that 50% were below-average yielders. In C-SSI, none of the below-average yielding entries were enlisted among the top 10.

**TABLE 6:** Number of genotypes with high (>3,000 kg/ha), below (<2,557 kg/ha), and above-average (>2,557–≤3,000 kg/ha) grain yield among top ten entries selected through various stability models.

S. No	SSI		Grain yield (kg/ha)		
	
			Below average (<2557)	Above average (>2557 ≤ 3000)	>3000
1.	NP-SSI	ASTAB	2	4	4
2.		MASV	2	5	3
3.		MASI	1	4	5
4.	P-SSI	ASTAB	5	4	1
5.		MASV	5	2	3
6.		MASI	5	3	2
7.	C-SSI	ASTAB	0	6	4
8.		MASV	0	7	3
9.		MASI	0	5	5
10.	BLUP	HMGV	0	3	7
11.		RPGV	0	3	7
12.		HMRPGV	0	3	7

*SSI, Simultaneous Selection Index; NP-SSI, Non-parametric Simultaneous Selection Index; P-SSI, Parametric Simultaneous Selection Index; C-SSI, SSI with culling strategy; ASTAB, AMMI-based stability parameter; MASV, modified AMMI stability value; MASI, Modified AMMI Stability Index; HMGV, harmonic mean of genotypic values; RPGV, relative performance of genotypic values; HMRPGV, harmonic mean of relative performance of genotypic values.*

The results of HMGV, RPGV, and HMRPGV established that none of the entries were below-average yielders. All the seven test entries with mean grain yields of more than 3,000 kg/ha were included in the top ten, which were relatively similar to the ranking based on mean grain yield alone. Similarly, poor grain yielders, such as Birsa Marua 1, ML 365, Shakti, PRM 1, and GN 4, were identified to be poor yielders with low stability.

## Discussion

The vagaries of weather conditions were observed, during the six rainy seasons, where the experimental material was tested. The highest rainfall was recorded during October 2013 (403 mm), whereas higher temperatures were recorded during July 2015 (Figure 1). The recorded precipitation and temperatures were found to be different every year and ultimately had an impact on the grain yield of the studied genotypes.

Combined ANOVA (Table 1) revealed that all components of variation, namely, environment (year), genotype, and GEI, were not only significant but can also be noticed through the percent sum of squares that the impact of environment (7.1%) was minimal compared to genotypes (51.8%) and GEI (37.8%). Although the environment component contributed less, an ample variation was explained by genotype interaction and the respective year. However, less, the variation due to the environment, including differences in rainfall and temperature, led to inconsistent performance of finger millet genotypes in the North Coastal Region of the state. In contrast to our results, a large proportion of total variation contributed by the environment was reported in finger millet in the studies by Adugna et al. (2011), Molla et al. (2013), Dagnachew et al. (2014); Birhanu et al. (2016), Lakew et al. (2017), and Seyoum et al. (2019) whereas the studies by Dehghani et al. (2006), Tolessa et al. (2013), and Singamsetti et al. (2021) unraveled GEI among the genotypes of field pea, wheat, and maize, respectively.

The procedure of AMMI included the partitioning of GEI by PCs, followed by the significance of the interaction was measured by Gollob’s *F*-test. F-statistics was used to identify the actual number of PCs to be considered for each axis of the testing mean square with an estimated error. Mean squares of AMMI analysis showed that, among GEI, four PCs recorded significance, and the first two PCs explained almost 75% of GEI with 124 corresponding degrees of freedom, indicating that most variation was captured by the first two components for grain yield (Table 2). Sood et al. (2017) and Mamo et al. (2018) recorded almost 90% of GEI contributed by the first two PCs in finger millet. As the first four PCs were significant (*p* < 0.001), AMMI4 was the best fit AMMI model for these multiyear yield trial data, explaining 97.5% of GEI. A significant proportion solely assures the phenotypic stability of genotypes over locations/years of GEI (Farshadfar and Sutka, 2006; Gauch, 2013). In AMMI, the visualization of the best stable genotype was provided by the biplots, and the genotypes to the extreme right and nearer to the axis were 34 (Indaf 9) and 1 (A 404), indicating that they were highly stable with more grain yield. In AMMI2, the most widely adopted genotype with more than mean grain yield was genotype 52 (RAU 8), indicated by its position almost at the origin point. Other genotypes nearer to the origin were 22 (GPU 45) and 51 (RAU 3), which were also highly stable across the environments. The high grain yielding genotypes were different in each year as shown by AMMI1 and 2 biplots, which suggested the impact of year-after-year variation of rainfall pattern and temperature during the crop period on the grain yield of finger millet genotypes. The amount of grain yield variance due to the genotype-by-year (i.e., GEI) effect, which is obtained from the MET data, played a key role in the identification of stable genotypes.

The stability parameters, namely, ASI, ASV, ASTAB, AVAMGE, DA, DZ, EV, FA, MASI, MASV, SIPC, and Za, were computed to compare whether they were equally efficient in assessing the stability of genotypes (Table 2). Among the genotypes, very low scores and less variation among different genotypes were recorded for parameters, such as EV and Za, deliberating that they might not be of much use in further calculations of SSI. The lower the score, the more stable a genotype is in any stability parameter and *vice versa*. As per ASI and ASV estimates, the genotypes viz., 55 (VL 146) followed by 45 (Paiyur 1) are the most stable while 57 (VL 324) followed by 22 (GPU 45) are according to all the other stability parameters except MASI and EV. In MASI and EV, genotype 57 (VL 324) was followed by 55 (VL 146). These differences depict the variation in estimation methods, whether they consider the first two or all the significant PCs. However, as a whole, all the stability parameters almost displayed a similar trend in identifying stable genotypes. Similar results were reported in the study by Cheloei et al. (2020) in rice using the same set of stability indices.

The association between all stability parameters was positively significant, implying that highly stable genotypes remained the same in almost all cases, whatever the index may be, indicating subtle differences in the calculation (Figure 2). Among all the significant associations, ASI and ASV that were computed based on the scores of the first two PCs were strongly correlated (*r* = 1), implying a similar trend in assessing the stability, though MASI is based on all four PCs but was extremely correlated (*r* = 0.99) with ASI and ASV, and hence a similar genotype ranking pattern. It might be due to capturing the maximum portion of GEI by the first two PCs. Comparatively weak but significant associations showed by MASV with ASI, ASV, and MASI due to their differences in weights assigned to various PCs in the computation of ASV and MASI. The stability parameters, such as ASTAB, AVAMGE, and Za, were highly significant, with all the remaining parameters implying almost similar calculations. A similar association between all these stability parameters was studied earlier by Ajay et al. (2020) in peanut and Sabaghnia et al. (2013) in wheat.

### Simultaneous Selection for Yield and Stability

Stable genotypes ensure sustainable yields, without much variation, every year. At the same time, it is well known that breeders or farmers prefer a cultivar with a high yield and average stability and not a highly stable genotype with just an above-average yield. In the quench to select the highest yielders, genotype selection simply based on mean grain yield in evaluation trials will mislead the plant breeders to select the wrong genotype that might not sustain over time due to its poor stability. Hence, the identification of a high-yielding and stable performing genotype is very much required for a plant breeder so that the cultivar can survive longer in the fields of the farmer.

### Ranking of Genotypes

Predominantly, two different attempts were made to identify stable high-yielding genotypes, such as (A) AMMI-based and (B) BLUP-based. In AMMI-based models, NP-SSI (Farshadfar, 2008; Ajay et al., 2020), P-SSI (Rao and Prabhakaran, 2007), and C-SSI were used. The culling strategy was earlier used in selection indices by Smith (1936) and Hazel (1943) while selecting genotypes for more than one economic trait, especially during the screening of disease-resistant genotypes. The utilization of this strategy in stability studies was not reported till date. The culling strategy used here is similar to the tandem culling proposed by Hazel and Lush (1942) where there was only one cutoff for one trait. Priority is to be given for the selection of high yielders only after culling for stability. In this study, we introduced this to ensure that the selected genotypes have at least an average or acceptable stability. All those genotypes with less than the mean stability score were considered as “stable genotypes.” Then, the highest yielder would be most advantageous among the qualified genotypes.

In the NP-SSI model, at least one or two out of top ten genotypes were with less than mean grain yield score and three to five genotypes (Indaf 9, A 404, PR 202, Sri Chaitanya, and GN1) recorded above 3,000 kg/ha grain yield. Early entry VL 324 (2,544 kg/ha) was at par with an average yield, and it was among the top ten ranks in all the methods studied. The SSI based on the ranking of the genotypes (i.e., NP-SSI) for both mean performance (either grain yield or any other trait) and stability may have inherent arbitrariness in the scoring procedure and were mostly biased toward the relative performance genotypes rather than their real worth. This arbitrariness is taken care of in other methods where actual values are being utilized to calculate the selection scores. A parametric approach, such as P-SSI, would be better when the data follow a normal distribution. Both rank-based and SSI selection methods may serve the purpose of selecting stable high yielders based on the population studied. Stable and high-yielding genotypes were identified through NP-SSI in the studies by Bose et al. (2014) in rice, Rea et al. (2020) in sugarcane, and Alizadeh et al. (2021) in rapeseed.

In all the three methods of the P-SSI, the top most genotype was VL 324 (2,544 kg/ha), which is an early entry. Another early entry, VL 146 (2,326 kg/ha) was also among the top ten in ASTAB and MASI. The genotypes were chosen among the top ten with more than 3,000 kg/ha grain yield varied with the stability parameters, namely, Indaf 9 in ASTAB, A 404 in MASI, as well as Sri Chaitanya, PR 202, and A 404 in MASV. In the SSI proposed by Smith (1936), weights were assigned to all the traits under consideration, to assess the real value of a genotype. However, there is a possibility that a higher value of another trait may compensate those with less value. Similarly, higher stability (low score of stability) may compensate for the low value of grain yield, resulting in a higher SSI score compared to other better performing genotypes. More stability cannot be compensated for low yield since the chief motive is higher yield. Grain yield is to be given more criteria compared to stability, but a certain level of stability expression is required. Although grain yield (70%) was given more weight compared to stability (30%), almost 50% of the top ten genotypes were below-average yielders irrespective of the stability parameter chosen (Table 6). The compensation of one factor over another was much more pronounced in P-SSI; hence, below-average yielders were observed among the top 10 ranks.

In the C-SSI approach, three to five genotypes (Indaf 9, A 404, Sri Chaitanya, PR 202, and GN1) were selected among the top ten with more than 3,000 kg/ha. By considering a separate cutoff value for early entries, VL 324 stood among the top stable high-yielding varieties. This method has shown that none of the top ten genotypes in ASTAB, MASV, and MASI were below-average yielders (Table 6). C-SSI takes advantage of NP-SSI and assures that none of them were below stability and below-average yielders. Compared to NP-SSI and P-SSI, this approach was more beneficial as it consisted of all the top ten genotypes with above-average grain yields.

The restricted maximum-likelihood (REML)/BLUP models emerged as the most acceptable procedure for genetic evaluation in breeding (Henderson, 1975), especially in GEI studies (Pires et al., 2011). The chief advantage of biometric approaches, such as HMGV, RPGV, and HMRPGV, is to disclose the randomness of the genotypic effects and to allow ranking genotypes in relation to their performance based on the genetic effects (de Resende et al., 2001). The BLUP-based simultaneous selections, such as HMGV, RPGV, and HMRPGV estimates, showed that seven entries (Indaf 9, A 404, Sri Chaitanya, PR 202, GN1, L5, and Bharathi) among the top 10 ranks had more than 3,000 kg/ha grain yield, while three entries were with above-average and none with below-average yield. It was also observed that the ranking of early entries was very high similar to that of grain yield ranks alone. The evaluation of adaptability and multi-trait stability of wheat genotypes through these BLUP-based indices was reported by Szareski et al. (2018). The estimates of HMGV, RPGV, and HMRPGV had the same genotype ranking that was reported in macaw palm by Rosado et al. (2019) and in Jatropa by Alves et al. (2018). Although the studied stability parameters were applied to various crops to estimate the stability and adaptability, no study was reported in finger millet crops. Bharathi and L5 recorded more than the mean stability scores (Table 3) of ASTAB, MASV, and MASI, indicating that they are less stable, and hence culled out in C-SSI while they were included in the top ten in the BLUP-based SSI. To ensure the selection of only stable genotypes, C-SSI might prove to be a better approach.

## Conclusion

Various stability parameters, such as ASV, ASTB, AVAMGE, DA, DZ, EV, and FA, considered in this study proved that all are equally potential in the identification of stable genotypes. All of the SSI models were almost similar in identifying the stable high-yielding genotypes; any one of these can be an alternative approach. Nevertheless, less stable genotypes with more yield or *vice versa* may be selected by rank-based NP-SSI, with more chance for the compensation of stability score for high yield in P-SSI, whereas in C-SSI and BLUP-based methods, there is no possibility to select low yielders. Hence, the last two methods may be best for selecting stable high-yielding genotypes. To ensure high stability, the C-SSI method can be suggested as the best approach. To conclude, medium-to-late varieties, namely, Indaf 9, Sri Chaitanya, PR 202, and A 404, whereas early entries, namely, VL 324 and VL 146, can be decisively considered as stable high-yielding genotypes for finger millet cultivation.

## Data Availability Statement

The original contributions presented in the study are included in the article/Supplementary Material, further inquiries can be directed to the corresponding authors.

## Author Contributions

NA and AS contributed to the conceptualization and designing of the study, field experimentation, and preparation of the manuscript. NA, NG, and TL contributed to data analysis. NA, YS, and UT performed field experiment. TP, AN, and VT edited the manuscript. All authors have read and approved the final manuscript.

## Conflict of Interest

The authors declare that the research was conducted in the absence of any commercial or financial relationships that could be construed as a potential conflict of interest.

## Publisher’s Note

All claims expressed in this article are solely those of the authors and do not necessarily represent those of their affiliated organizations, or those of the publisher, the editors and the reviewers. Any product that may be evaluated in this article, or claim that may be made by its manufacturer, is not guaranteed or endorsed by the publisher.
