# Supplementary material for: Comparative Study of AMMI- and BLUP-Based Simultaneous Selection for Grain Yield and Stability of Finger Millet [Eleusine coracana (L.) Gaertn.] Genotypes
Source: Front Plant Sci. 2022 Jan 6;12:786839. doi: 10.3389/fpls.2021.786839 (PMC8770906; doi:10.3389/fpls.2021.786839)
Supplement: Supplementary file 1 [file Data_Sheet_1.docx]

**Supplementary Table 1. Within-environment analysis and descriptive statistics**

| **Year** | **Genotypes** | | **Replication** | | **Error** | | **Mean** | **Range (kg/ha)** | | **LSD** | **CV** | **H** |
| --- | --- | --- | --- | --- | --- | --- | --- | --- | --- | --- | --- | --- |
|  | *df* | MSS | *df* | MSS | *df* | MSS |  | Maximum | Minimum |  |  |  |
| 2011 | 59 | 90.6^***^ | 1 | 13.8^ns^ | 59 | 5.53 | 2714 | 1310 | 4001 | 4.56 | 8.67 | 93.9 |
| 2012 | 59 | 75.9^***^ | 1 | 8.55^ns^ | 59 | 3.7 | 2425 | 1093 | 3916 | 3.75 | 7.93 | 91.1 |
| 2013 | 59 | 87.9^***^ | 1 | 1.07^ns^ | 59 | 1.05 | 2329 | 645 | 3580 | 2.03 | 12.39 | 89.8 |
| 2014 | 59 | 55.3^***^ | 1 | 3.97^ns^ | 59 | 5.68 | 2668 | 1404 | 3990 | 4.52 | 8.93 | 79.7 |
| 2015 | 59 | 41.6^***^ | 1 | 5.42^ns^ | 59 | 3.12 | 2610 | 1215 | 3541 | 3.39 | 11.76 | 92.5 |
| 2016 | 59 | 38.7^***^ | 1 | 2.79^ns^ | 59 | 3.28 | 2598 | 1455 | 3675 | 3.5 | 10.97 | 91.5 |

***, significant at 0.1(p<0.001); ns, non-significant; *df*, degrees of freedom; MSS, mean sum of squares; LSD, least significant difference; CV, co-efficient of variation; H, heritability (broad sense)

**Supplementary Table 2. Top ten entries identified through the minimum qualified cutoff values**

| **Rank** | **NP-SSI** | | | **P-SSI** | | | **C-SSI** | | | **BLUP** | | |
| --- | --- | --- | --- | --- | --- | --- | --- | --- | --- | --- | --- | --- |
|  | **ASTAB** | **MASV** | **MASI** | **ASTAB** | **MASV** | **MASI** | **ASTAB** | **MASV** | **MASI** | **HMGV** | **RPGV** | **HMRPGV** |
| 1 | Indaf 9  (3419⁋) | Sri chaitanya  (3251) | Indaf 9  (3419) | VL 324  (2544) | VL 324  (2544) | VL 324  (2544) | Indaf 9 (3419) | Indaf 9  (3419) | Indaf 9  (3419) | Indaf 9  (3419) | Indaf 9  (3419) | Indaf 9  (3419) |
| 2 | A 404  (3374) | GPU 48  (2791) | A 404  (3374) | GPU 45  (2524) | GPU 45  (2524) | VL 146 (2326) | A 404  (3374) | Sri chaitanya  (3251) | A 404  (3374) | A 404  (3374) | A 404  (3374) | A 404  (3374) |
| 3 | GPU 48  (2791) | PR 202  (3230) | Sri chaitanya  (3251) | RAU 3 (2599) | GPU 48 (2791) | RAU 3  (2599) | Sri chaitanya  (3251) | PR 202 (3230) | Sri chaitanya  (3251) | Sri chaitanya  (3251) | L 5 (3242) | Sri chaitanya  (3251) |
| 4 | CO 13  (2740) | CO 13  (2740) | CO 13  (2740) | ML 365 (1623) | ML 365 (1623) | CO 13  (2740) | PR 202  (3230) | KMR 204 (2960) | PR 202  (3230) | PR 202  (3230) | Sri chaitanya  (3251) | L 5 (3242) |
| 5 | PR 202  (3230) | Indaf 9  (3419) | GPU 48  (2791) | GPU 48  (2791) | Sri chaitanya  (3251) | ML 365 (1623) | Poorna (2948) | Poorna (2948) | GN 1 (3016) | L 5 (3242) | PR 202  (3230) | PR 202  (3230) |
| 6 | Sri chaitanya  (3251) | Paiyur 1 (2925) | PR 202  (3230) | VL 146 (2326) | PR 202  (3230) | GPU 48  (2791) | HR 911 (2875) | Paiyur 1 (2925) | GPU 67 (2822) | Bharathi (3119) | Bharathi (3119) | Bharathi (3119) |
| 7 | RAU 3  (2599) | Poorna (2948) | RAU 3  (2599) | CO 13  (2740) | CO 13  (2740) | GPU 45  (2524) | Indaf 8 (2851) | RAU 8 (2899) | GPU 48  (2791) | GN 1 (3016) | GN 1 (3016) | GN 1 (3016) |
| 8 | VL 324  (2544) | VL 324  (2544) | VL 324  (2544) | Hamsa  (2613) | K 7 (2429) | Indaf 9  (3419) | GPU 67 (2822) | HR 911 (2875) | CO 13  (2740) | GPU 28 (2988) | GPU 28 (2988) | GPU 28 (2988) |
| 9 | GPU 45  (2524) | GPU 45  (2524) | GN 1 (3016) | CO 12 (2168) | Indaf 9  (3419) | Hamsa  (2613) | GPU 48  (2791) | GPU 67 (2822) | CO 10 (2708) | Paiyur 1 (2925) | KMR 204 (2960) | Paiyur 1 (2925) |
| 10 | Hamsa  (2613) | RAU 8 (2899) | Hamsa  (2613) | Indaf 9  (3419) | MR 6 (2566) | A 404 (3374) | CO 13  (2740) | GPU 48  (2791) | Hamsa  (2613) | Poorna (2948) | Paiyur 1 (2925) | Poorna (2948) |

⁋ Grain yield in kg/ha
